# Did the Ambient Ozone Affect Stem Increment of Scots Pines (*Pinus sylvestris* L.) on Territories under Regional Pollution Load? Step III of Lithuanian Studies

**DOI:** 10.1100/tsw.2007.55

**Published:** 2007-03-21

**Authors:** Algirdas Augustaitis, Ingrida Augustaitiene, Gintautas Cinga, Juozapas Mazeika, Romualdas Deltuvas, Romualdas Juknys, Adomas Vitas

**Affiliations:** ^1^ Lithuanian University of Agriculture, LT-53362 Kaunas dstr., Lithuania; ^2^ Vytautas Magnus University, LT-44404 Kaunas, Lithuania

**Keywords:** ambient ozone, peak concentration, basal area increment

## Abstract

This study aimed to explore if changes in stem increment of Scots pines (*Pinus sylvestris* L.) could be related to changes in ambient ozone concentration when the impact of tree dendrometric parameters (age, diameter) and crown defoliation are accounted for. More than 200 dominant and codominant trees from 12 pine stands, for which crown defoliation had been assessed since 1994, were chosen for increment boring and basal area increment computing. Stands are located in Lithuanian national parks, where since 1994–95 Integrated Monitoring Stations have been operating. Findings of the study provide statistical evidence that peak concentrations of ambient ozone (O_3_) can have a negative impact on pine tree stem growth under field conditions where O_3_ exposure is below phytotoxic levels.

## INTRODUCTION

Relationships between ambient ozone ($O_3$) and tree growth, focusing on physiological or biochemical effects on tree seedlings, have been extensively documented in artificial conditions[1,2,3,4]. However, these data do not apply well to actual forest conditions and growth of large trees[5,6,7,8]. Due to chamber effects, extrapolation of these results to trees in the forest is impossible[7]. Much is written about phytotoxic effect of $O_3$ on trees in forests with elevated concentrations[9,10,11], however, too little is known about the $O_3$ effect on a regional scale, where this effect is subtle and difficult to detect, or often fails[12,13] despite sophisticated approaches[14], and interaction of $O_3$ exposure with air temperature and global radiation[15].

Forest monitoring usually concentrates on nonspecific response indicators, i.e., tree defoliation and tree growth, which are subject to many stressors other than $O_3$ and therefore its causative impact on them is difficult to generalize[13]. However, due to expected increase in ambient $O_3$ concentration in the future[15], it is necessary to determine if exposure to ambient levels of $O_3$ actually affect tree growth in natural forests[7]. Therefore, in our final step of the Lithuanian studies, we attempted to investigate possible relationships between tree growth and peak $O_3$ concentrations in order to quantify its contribution to the integrated impact of other key factors on pine stem increment.

The findings of the previous parts of our study allow us to make an assumption that changes in temporal and spatial changes in pine defoliation most reliably could be related to acidifying compounds and meteorology, whereas the contribution of peak $O_3$ is smaller but significant ($p < 0.05$). Ozone reinforced the integrated impact of other key factors by only 1%. Therefore, in the presented study, we attempted to investigate the possible direct effect of peak $O_3$ concentration on pine stem basal increment after the influence of crown defoliation had been accounted for. The effect of different acidifying compounds on foliage is well known[16,17]. Separation of the direct effect of $O_3$ on tree growth from the effect of acidifying compounds on foliage was accomplished by means of correlative analysis. Reduction in productivity due to disturbances in transpiration, carbohydrate metabolism and movement, and mineral nutrient deficiencies[18,19,20] seemed to be the most relevant, since changes in growth often occur at the level below the ambient air quality standards[21] or may proceed without signs of the usual leaf damage[22]. Findings of the study should help to understand the $O_3$ effect and contribution of peak values to the changes in pine stem basal area increment.

## MATERIALS AND METHOD

To pursue the objectives of the present study, we chose 12 out of 48 pine permanent observation stands (POS) and examined their defoliation changes in detail (Step II). More than 200 dominant and codominant trees, for which crown defoliation had been assessed since 1994, were chosen for the increment boring according to the third stage of the methodology presented in Step II of this study. Characteristics of POS are compiled in Table 1.

A standard dendrochronological technique was used to assess tree growth rates. Radial growth was assessed by measuring the width of annual rings in stem cores. Each ring was measured to the closest 0.01 mm using an electronic transducer and binocular scope fixed over the moving stage. The obtained data series on tree increment are presented in Fig. 1. Based on ring widths and measured diameter, we computed diameter for each year of the considered period, then stem basal areas, and finally stem basal area increment (BAI). For each POS, a BAI chronology was computed by averaging the BAI for each year across all trees sampled at that POS. In such a way, mean defoliation of sample trees was computed for each year.

Concentrations and deposition of the considered pollutants were obtained from the Integrated Monitoring Stations (IMS) and are presented in Steps I and II of our studies. Correlation analysis was employed to test the relationship between tree growth and the pollution data over the period from 1994 to 2003, multiple regression analysis was used to quantify contribution of peak $O_3$ concentrations to tree BAI increment residuals and to detect timber losses.

The model of timber losses was created according to the hypothesis that tree stem growth is the function of foliage biomass or defoliation and productivity of foliage, i.e., of assimilation intensity and ability to allocate carbon. The obtained losses could be evaluated as the highest, which possibly could have occurred due to the recently observed increase in peak $O_3$ concentration in the prevailing Lithuanian pine stands. The value of the timber increment losses was calculated by employing a model developed for that purpose. For calculation of an average price for pine timber, local assortment tables and current prices for pine assortments were used. All Lithuanian pine stands older than 40 years were selected from the stand-wise forest inventory database to determine total timber increment losses.

**TABLE 1**
**Characteristics of the Considered POS**

| POS | Stand and Site Parameters | | | | | | |
|---|---|---|---|---|---|---|---|
| | **Age (year)** | **Mean Diameter (cm)** | **Mean Height (m)** | **Sum of Basal Area ($m^2 \cdot ha^{-1}$)** | **Volume ($m^3 \cdot ha^{-1}$)** | **Tree Density ($unit \cdot ha^{-1}$)** | **Forest Type*** |
| Aukstaitija National Park | | | | | | | |
| ANP-1 | 170 | 41.4 | 29.5 | 31,0 | 417 | 221 | Vac-myr |
| ANP-2 | 60 | 18.5 | 19.7 | 17,2 | 173 | 600 | Vac-myr |
| ANP-3 | 170 | 42.4 | 31.0 | 32,0 | 456 | 220 | Vac-myr |
| ANP-4 | 60 | 17.8 | 19.4 | 17,5 | 175 | 622 | Vac-myr |
| ANP-5 | 170 | 41.7 | 29.9 | 27,8 | 381 | 194 | Vac-myr |
| ANP-6 | 60 | 16.6 | 17.1 | 19.6 | 177 | 772 | Vac-myr |
| Zemaitija National Park | | | | | | | |
| ZNP-1 | 110 | 392 | 289 | 26,0 | 301 | 134 | Myr-ox |
| ZNP-2 | 110 | 379 | 282 | 35,9 | 402 | 198 | Myrtil |
| ZNP-3 | 100 | 315 | 248 | 25,8 | 264 | 152 | Myrtil |
| Dzukija National Park | | | | | | | |
| DNP-1 | 50 | 17.3 | 17.4 | 27.2 | 246.4 | 1156 | Clad |
| DNP-2 | 70 | 26.1 | 17.3 | 22.2 | 200.8 | 413 | Clad |
| DNP-3 | 70 | 29.9 | 20.9 | 31.0 | 307.8 | 442 | Vac |

* Vac-myr, *Pinetum vacciniosum-myrtilosum*; Myr-ox, *P. myrtilosum-oxalidosum*; Myrtil, *P. myrtilosum*; Clad, *P. cladoniosum*; Vac, *P. vacciniosum*.

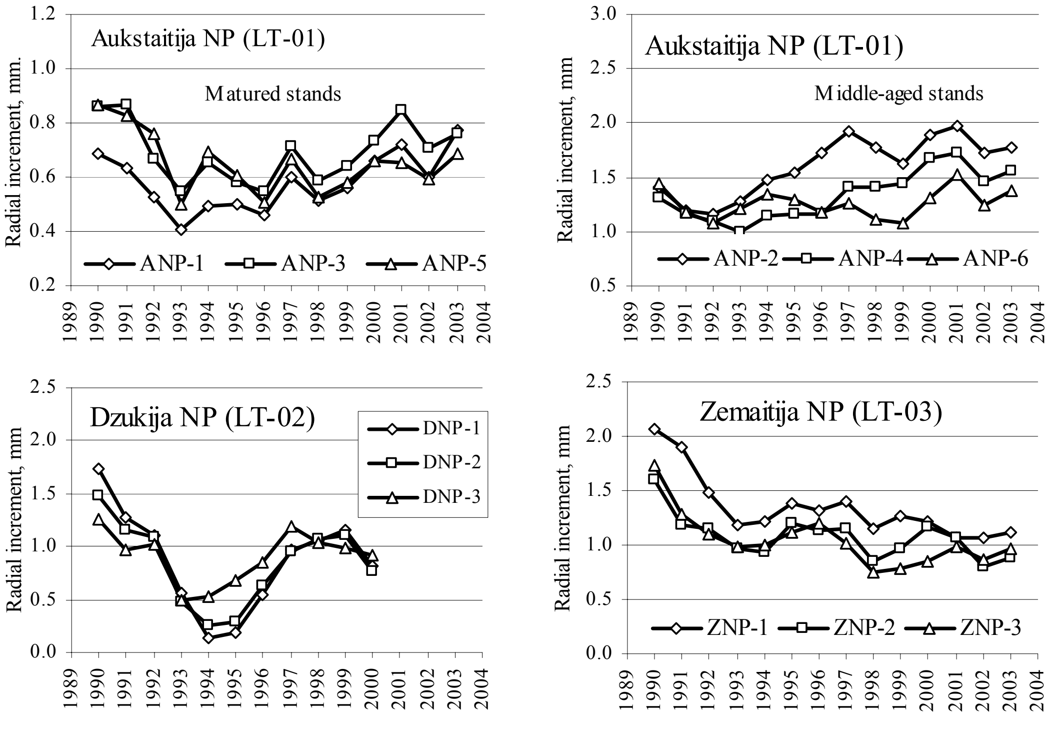

**FIGURE 1.** Radial increment of pine trees at different sites.

## RESULTS

Contribution of the $O_3$ effect to the changes in residual increment was quantified after the influences of tree dendrometric parameters (age, diameter) and crown defoliation had been accounted for. Correlation between pine stem BAI and crown defoliation was strongest (r = –0.512), followed closely by positive correlation with tree diameter (r = 0.382), and a weaker negative correlation with stand age (r = –0.081). Integrated impact of these parameters was analyzed by the means of multiregression model:

$$Zq = 7.330 - 0.189 \times F - 0.088 \times A + 0.50 \times D;\ R^2 = 0.795,\ p < 0.05 \qquad (1)$$

Crown defoliation (*F,* %), tree age (*A,* years), and diameter (*D,* cm) accounted for 80% of spatial and temporal variability in pine stem BAI (*Zq,* $cm^2$). Correlation analysis between part of BAI residuals and considered predictor variables revealed the highest significance of peak $O_3$ impact on increment ($p < 0.001$) (Fig. 2). These findings indicate a direct effect of ambient $O_3$ on changes in pine stem increment, probably due to disturbances in $CO_2$ assimilation and carbohydrate movement within the trees.

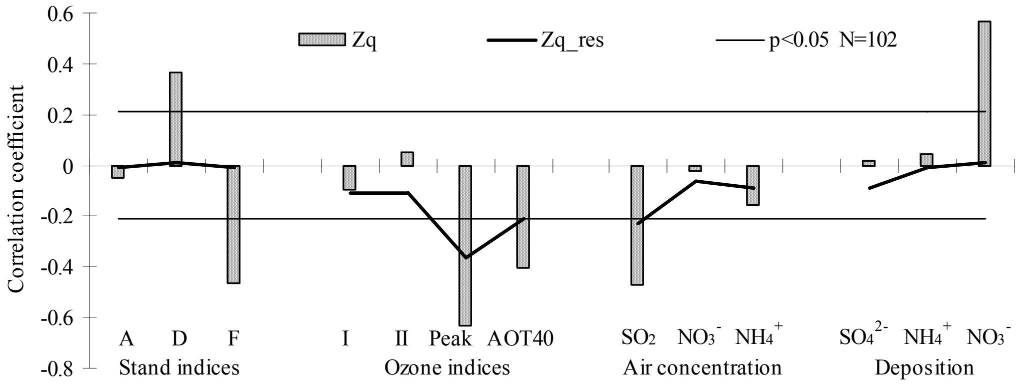

**FIGURE 2.** Relationships between basal area (Zq), and its residuals (Zq_res) with considered parameters of stand and pollution (I, mean value of $O_3$ for April–August; II, annual mean value of $O_3$).

The obtained results revealed that after the effect of defoliation was accounted for, the $SO_2$ effect on residuals decreased substantially (Fig. 2), whereas the integrated impact of all air acidifying compounds accounted for only 6.3% of BAI residual variability (Model F-1), and their deposition for 2.5% (F-3) (Table 2). Thus, we can conclude that the key factor contributing to increment residual changes could be peak $O_3$ concentrations while $SO_2$ only reinforced the $O_3$ effect on stem increment.

Special emphasis was given to the integrated impact of nitrogen and $O_3$ on pine stem growth. Both pollutants are known to affect growth and biomass. Ozone has negative impact on growth, whereas nitrogen has positive fertilization effect[23]. In this study, no significant effects of air concentrations of $NO_3^-$ and $NH_4^+$, and deposition on BAI residuals, were detected ($p > 0.05$) (F-5). However, their integrated impact with peak $O_3$ value seemed to have synergetic effect. The sum of their effects was greater than individual.

Integrated impact of air acidifying compounds and their deposition accounted for 18.5% of variability in stem increment residual (Model F-4). Ozone increased the degree of the explanation by 8.7% up to 27.2%. Integrated impact of meteorological parameters accounted for 10.2% of variability of stem increment residual (F-8), and $O_3$ increased this rate up to 17.2% (by 7.0%). Integrated impact of air acidifying compounds, acid deposition, and meteorological parameters accounted for up to 21% of variability in residual of stem BAI (F-9). Ozone increased this rate of explanation by approximately 10% up to 31.6%.

**TABLE 2**
**Contribution of Ambient $O_3$ to the Integrated Impact of Different Environmental Factors on Residual of Pine Stems BAI**

| Variables | Models, F(a.b) | | | | | | | | |
|---|---|---|---|---|---|---|---|---|---|
| | **1(3.98)** | **2(3.98)** | **3(3.98)** | **4(4.97)** | **5(4.97)** | **6(2.99)** | **7(4.97)** | **8(4.97)** | **9(8.93)** |
| In the air: | | | | | | | | | |
| $SO_2$ | + | | | + | | | | | – |
| $\Sigma NH_4^+$ | – | | | | – | | | | |
| $\Sigma NO_3^-$ | – | | | + | – | | | | + |
| In precipitation (P): | | | | | | | | | |
| $SO_4^{2-}$ | | – | | | | | | | |
| $NH_4^+$ | | – | | | – | | | | |
| $NO_3^-$ | | – | | | – | | | | |
| Deposition (D): | | | | | | | | | |
| $SO_4^{2-}$ | | | – | | | | | | + |
| $NH_4^+$ | | | – | + | | | | | |
| $NO_3^-$ | | | – | + | | | | | + |
| Precipitation (Pr): | | | | | | | | | |
| Last year: | | | | | | | | | |
| IX–XI | | | | | | | | | |
| XII–II | | | | | | | | | |
| Current year: | | | | | | | | | |
| III–V | | | | | | – | | – | |
| VI–VIII | | | | | | – | | – | – |
| Temperature (Tm): | | | | | | | | | |
| Last year: | | | | | | | | | |
| IX–XI | | | | | | | + | + | + |
| XII–II | | | | | | | – | | – |
| Current year: | | | | | | | | | |
| III–V | | | | | | | + | + | |
| VI–VIII | | | | | | | – | | – |
| $r^2$ , % | 6.3 | 0.4 | 2.5 | 18.5 | 0.7 | 0.2 | 7.7 | 10.2 | 21.7 |
| $r^{2*}$ with $O_3$ effect, % | 15.3 | 16.4 | 19.4 | 27.2 | 23.9 | 14.4 | 17.2 | 17.2 | 31.6 |
| $O_3$ effect ($r^{2*} – r^2$), % | 9.0 | 16.0 | 16.9 | 8.7 | 23.2 | 14.2 | 9.5 | 7.0 | 9.9 |
| $O_3$ significance: $p <$ | 0.002 | 0.000 | 0.000 | 0.001 | 0.000 | 0.000 | 0.001 | 0.005 | 0.000 |

*Note:* individual impact of peak $O_3$ concentration on stem basal area increment residual: $r^2$=13.3% and $p$<0.0002.

F(a.b) - models identified by F - test symbol with degrees of freedom: a - of the predictor variables; b - of the observations (STATISTICA software); significance of variables (+), $p$<0.05 and (–), $p$>0.05 in model.

**F1(3.98) =** $0.501 – 0.800 \times SO_2 + 0.306 \times \Sigma NO_3^- + 0.163 \times \Sigma NH_4^+$;
**F2(3.98) =** $0.463 – 0.418 \times P\_SO_4^{2-} + 0.234 \times P\_NH_4^+ – 0.693 \times P\_NO_3^-$;
**F3(3.98) =** $0.326 – 0.0031 \times D\_SO_4^{2-} + 0.0029 \times D\_NH_4^+ + 0.0003 \times D\_NO_3^-$;
**F4(4.97) =** $0.749 – 1.823 \times SO_2 + 3.546 \times \Sigma NO_3^- + 0.0062 \times D\_NH_4^+ – 0.0092 \times D\_NO_3^-$;
**F5(4.97) =** $0.312 – 0.427 \times \Sigma NO_3^- – 0.205 \times \Sigma NH_4^+ + 0.518 \times P\_NH_4^+ + 0.125 \times P\_NO_3^-$;
**F6(2.99) =** 0.406 – 0.004 × Pr(III–V) + 0.0009 × Pr(VI–VIII);
**F7(4.97) =** –1.704 + 0.275 × Tm(IX–XI) – 0.042 × Tm(XII–II) – 0.366 × Tm(III–V) + O.132 × Tm(VI–VIII);
**F8(4.97) =** 2.371 – 0.005 × Pr(III–V) + 0.002 × Pr(VI–VIII) + 0.188 × Tm(IX–XI) – 0.510 × Tm(III–V);
**F9(8.93) =** $–8.042 – 1.743 \times SO_2 + 2.829 \times \Sigma NO_3^- + 0.0068 \times D\_SO_4^{2-} – 0.012 \times D\_NO_3^- + 0.004 \times$ Pr(VI–VIII) + 0.257 × Tm(IX–XI) – 0.295 × Tm(XII–II) + O.320 × Tm(VI–VIII).

Finally, we attempted to evaluate the economic losses due to increase in peak $O_3$ concentration from 125 to 165 $\mu g \cdot m^{-3}$ and mean defoliation from 18.3 to 20.9%, what was approximately monitored over 2001–2003 period. In this case, we computed stem basal area as sum of residual (ΔZq) and as a part of BAI, which was regressed with defoliation, age, and diameter of pine stands (Eq. 1):

$$Zq = \Delta Zq + [7.330 - 0.189 \times F - 0.088 \times A + 0.50 \times D] \quad (2)$$

Defoliation and residuals were the function of peak $O_3$ concentration:

$$F = 12{,}137 \times e^{0.0033 \times O_3}; r = 0.288 \quad (3)$$

$$\Delta Zq = 3.829 - 0.025 \times O_3; r = 0.364 \quad (4)$$

Economic evaluation of pine increment changes was computed developing the model of BAI in relation to peak $O_3$ concentration, mean tree age, and diameter:

$$Zq = 11.16 - 2.294 \times e^{0.003 \times O_3} - 0.088 \times A + 0.50 \times D - 0.025 \times O_3; R^2 = 0.835, p < 0.05 \quad (5)$$

Reduction of wood growth due to the effect of ambient $O_3$ typically ranged from 3–22% in the U.S. and Europe as well[10,20,23,24]. In Lithuania, the increase of peak $O_3$ values could have resulted in pine annual increment decrease of approximately 0.73 $m^3$ $ha^{-1}$, what makes 12.7% of a total annual pine increment in Lithuania equivalent to 0.39 million $m^3$. When this decrease in the increment was valued at the average stumpage prices, the annual average losses would reach €16.1 $ha^{-1}$, and the total annual losses for Lithuania would be as high as €8.6 million.

## DISCUSSION

Numerous research studies underscore the negative effect of ambient $O_3$ on tree photosynthesis and biomass production during long-term exposure to moderately elevated $O_3$ concentration[25,26] and regionally elevated concentration as well[23]. However, the detection of the negative ambient $O_3$ effect on mature trees under field conditions is more complicated than under artificial conditions, where experiments have not yet precisely determined if ambient $O_3$ levels actually impair it[7]. Despite this, we could conclude, that $O_3$ has a considerable negative impact on pine stem BAI in Lithuania.

In this study, we attempted to detect possible direct effect of $O_3$ on tree growth after the influence of defoliation on BAI was accounted for. To meet the objective, we simplified the complicated and insufficiently investigated effect of $O_3$ on trees[7,27] to its impacts on:

1. Mesophyll tissue[27] resulting in reduction of $CO_2$ assimilation[19], decrease in needle longevity, and premature foliar senescence as well as earlier loss of foliage – defoliation[28]
2. Plant metabolism and movement of carbohydrate[10,18,29,31,32], increase in respiration[19,33], disturbance in mineral nutrient deficiencies[18,34,35], and decrease in photosynthesis and productivity[18,19,35,36] – growth reduction[21,37]

Moreover, tree growth reduction could additionally occur due to decrease in foliage biomass caused by other environmental factors. Therefore, this procedure allowed the elimination of the impact of air concentration of acidifying compounds and their deposition on BAI, and verified strong interaction of acid compounds with crown defoliation that was detected not only in our earlier studies, but also in the cause-effect studies on pine defoliation changes in Europe[38]. In our opinion, elimination of the defoliation impact on tree increment was a good example of an attempt to separate the effects of different pollutants[30], i.e., acidifying compounds and ambient $O_3$ by empirical regression methods.

The detected effect of ambient $O_3$ on stem growth was in full agreement with the findings in other countries[14,23,24,35]. The detected significant effect of springtime highest $O_3$ concentration in Finland[35] and the Carpathian forest[14], a higher significance of mean diurnal $O_3$ concentration than accumulated (AOT40) in Sweden[23], could be the basis for a conclusion that changes in Scots pine stem growth could be related to changes in peak ambient $O_3$ concentration.

However, these results were obtained by regressing data on tree growth with $O_3$ concentrations and other environmental factors. Such methods can reveal stress effects on radial growth, but cannot conclusively prove causation[7]. It has been suggested that the predictor variable must be selected from the hypothetically adopted cause-effect relationships based on relevant literature information[27]. Then, correlation analysis can be used to assess the relative contribution of the predictor variable to the response variable; however, a significant relationship does not necessarily mean a causal relationship. We support this point of view concerning causation of the significant correlation. However, in some cases, when detailed and thorough studies are completed, indirect signs, such as a significant relationship, cannot be ignored even in cases when explanation of causation of the effect is difficult due to the present state of knowledge or knowledge gaps. In our study, peak $O_3$ concentration demonstrated higher relationships with all of the considered components of biota including pine defoliation and BAI than with such a widespread index as AOT40 or mean values of $O_3$ concentrations over vegetation period. The AOT40 is a widespread indicator that analyzes possible $O_3$-induced injury to plants, however, its limitations are well known[21,23,39]. Neither mean $O_3$ value for a vegetation period nor its annual concentration, in particular, showed significant effects on pine growth, which was in full agreement with the present knowledge. These results could be presented as a proof that correlative analysis reliably reflects effect of these parameters. Based on this assumption, can we ignore the detected significant effect of peak $O_3$ concentrations on the considered components of biota and pine defoliation and increment in particular? This is the question to be dealt with in our further studies in this field.

Data from the ICP IMS, where air pollutants have been continuously monitored[40,41] offered a possibility to get more insight into $O_3$ effect on tree growth. Peak $O_3$ concentration has more significant effect on tree defoliation and increment than other indices, such as AOT40, for vegetation and forest as well as mean $O_3$ concentrations for a vegetation period. Therefore, more thorough studies with continuous active monitors are needed, especially in the northern countries where $O_3$ concentrations very seldom reach the level of toxicity. However, impact of ambient $O_3$ on native forest ecosystems could be higher than in the southern countries where $O_3$ concentrations often exceed the phytotoxic level of the AOT index, but significant relations with tree damages fail[12].

## CONCLUSION

Changes in pine stem increment could, first of all, be related to tree parameters and crown defoliation. Peak $O_3$ concentrations had the most significant effect on the residual part of increment, when the integrated effect of these parameters was accounted. Ozone most intensively increased the effect of air acidifying compounds and acid deposition, followed closely by the effect of meteorological parameters. We estimate that the recent increase in the peak $O_3$ concentrations could result in a 12.7% decrease of a total annual pine increment in Lithuania.
